# Co-ordinated Role of TLR3, RIG-I and MDA5 in the Innate Response to Rhinovirus in Bronchial Epithelium

**DOI:** 10.1371/journal.ppat.1001178

**Published:** 2010-11-04

**Authors:** Louise Slater, Nathan W. Bartlett, Jennifer J. Haas, Jie Zhu, Simon D. Message, Ross P. Walton, Annemarie Sykes, Samer Dahdaleh, Deborah L. Clarke, Maria G. Belvisi, Onn M. Kon, Takashi Fujita, Peter K. Jeffery, Sebastian L. Johnston, Michael R. Edwards

**Affiliations:** 1 Department of Respiratory Medicine, National Heart & Lung Institute, Imperial College London, London, United Kingdom; 2 MRC & Asthma UK Centre in Allergic Mechanisms of Asthma, London, United Kingdom; 3 Centre for Respiratory Infection, London, United Kingdom; 4 Lung Pathology, National Heart & Lung Institute, Imperial College London, London, United Kingdom; 5 Imperial Healthcare NHS Trust, London, United Kingdom; 6 Respiratory Pharmacology, National Heart & Lung Institute, Imperial College London, London, United Kingdom; 7 Institute of Virus Research, Kyoto University, Kyoto, Japan; University of North Carolina at Chapel Hill, United States of America

## Abstract

The relative roles of the endosomal TLR3/7/8 versus the intracellular RNA helicases RIG-I and MDA5 in viral infection is much debated. We investigated the roles of each pattern recognition receptor in rhinovirus infection using primary bronchial epithelial cells. TLR3 was constitutively expressed; however, RIG-I and MDA5 were inducible by 8–12 h following rhinovirus infection. Bronchial epithelial tissue from normal volunteers challenged with rhinovirus *in vivo* exhibited low levels of RIG-I and MDA5 that were increased at day 4 post infection. Inhibition of TLR3, RIG-I and MDA5 by siRNA reduced innate cytokine mRNA, and increased rhinovirus replication. Inhibition of TLR3 and TRIF using siRNA reduced rhinovirus induced RNA helicases. Furthermore, *IFNAR1* deficient mice exhibited RIG-I and MDA5 induction early during RV1B infection in an interferon independent manner. Hence anti-viral defense within bronchial epithelium requires co-ordinated recognition of rhinovirus infection, initially via TLR3/TRIF and later via inducible RNA helicases.

## Author Summary

Host-pathogen interactions are mediated by pattern recognition receptors that identify conserved structures of micro-organisms that are distinct from self. During a viral infection, important pattern recognition receptors include the endosomal Toll-like receptors (TLRs), and a second set of cytoplasmic pattern recognition receptors known as the RNA helicases. Many studies have highlighted the importance of TLR3, TLR7/8 and the RNA helicases in providing robust anti-viral immunity via interferon induction and inflammation. Both endosomal TLR and cytoplasmic RNA helicase mediated pathways are believed to exist as separate yet non-redundant entities; however, little thought is given to why both systems exist, and few studies also consider how both pathways together contribute to anti-viral immunity. Using models of rhinovirus infection in primary bronchial epithelial cell culture *in vitro* and experimental infection in mouse and human models *in vivo*, we show that the RNA helicases are preferentially induced early in the infection cycle via TLR3 mediated signaling events, and work in a co-ordinated, systematic manner. The results help understand the complex events that determine effective innate immunity to rhinovirus infection and how these processes contribute to virus induced exacerbations of asthma and chronic obstructive pulmonary disease.

## Introduction

Human rhinovirus (RV) belongs to the *Picornaviridae* family and are implicated in an extensive range of human respiratory disorders including the common cold, viral bronchiolitis, and exacerbations of asthma and chronic obstructive pulmonary disease [1]–[5]. RV are classified as major or minor group based on receptor usage, or RNA identity as RV-A and RV-B. RVs of both major and minor groups are associated with human disease. Recently, this phylogeny been changed to include the newly designated RV-C group which represent a distinct group of RV [6]. RV of all groups generally infect the epithelial cells of both the upper and lower airway, and are responsible for the induction of a range of mediators including pro-inflammatory cytokines and growth factors [7]–[11], type I interferon (IFN)-β and type III IFN-λs [12]. Pro-inflammatory cytokines contribute to the duration and severity of RV induced illnesses [13]–[16]. Recently, primary human bronchial epithelial cells (HBECs) from asthmatics were found to be defective in IFN-β and IFN-λ mRNA and protein, [17], [18], providing a likely explanation for the increased vulnerability to virus induced asthma exacerbations and enhanced symptom severity observed [16], [19]. Understanding the mechanisms responsible for these deficiencies in asthma, as well as identifying new anti-inflammatory therapies requires a detailed understanding of the innate reponses to RV infection.

Much is now known about the signal transduction pathways utlised by viruses to induce cytokines and IFNs. RNA viruses are initially sensed through pattern recognition receptors (PRRs), such as recognition of dsRNA by endosomal Toll-like receptor (TLR)-3, [20], [21] or ssRNA by endosomal TLR7/8 [22], [23]. TLR3 utilises the adaptor TIR domain-containing adapter inducing IFN-β (TRIF), to activate IκB kinase (IKK-ι/ε) and TANK binding kinase-1 (TBK-1), and IKK-β activating interferon regulatory factor (IRF)-3 and NF-κB, transcription factors required for IFN-β gene expression. Within the intracellular compartment, exists a second set of PRRs, the RNA helicases, including retinoic acid inducible gene (RIG-I) [24], melanoma differentiation associated gene-5 (MDA5) [25], and the inhibitory protein LGP2 [26], [27]. The helicases signal via their caspase recruitment domains (CARD), to adaptor inducing interferon-β (CARDIF) [28], (also known as IPS-1, MAVS and VISA, [29]–[31]), and activate TBK1, IKK-ι/ε and IKK-β, and hence IRF3 and NF-κB. Both RIG-I and MDA5 have been implicated in IFN-α/β production in various model systems. MDA5 recognises high molecular weight dsRNA [32], while the specificity of RIG-I has been marked with controversy. While originally identified as a dsRNA binding helicase [24], RIG-I has recently been shown to bind low molecular weight dsRNA [32] and also 5′-triphosphorylated ssRNA [33], [34]. The 5′-triphosphorylated ssRNA binding preferences of RIG-I suggest it is unable to recognize Picornavirus infections [33], [35]; which do not synthesis 5′-triphosphorylated RNA molecules. The relative importance of TLR3, MDA5 or RIG-I in viral infections has been partly defined by cells derived from *TLR3^−/−^* [21], *RIG-1^−/−^* or *MDA5^−/−^* mice [35], [36], however the importance of each PRR, including their exclusive or redundant roles in various infection models, and their direct relevance to human disease remains a subject of much debate.

In order to understand the recognition of RV infection, and the induction of both pro-inflammatory cytokines and IFNs, we investigated the role of TLR3/7/8, RIG-I and MDA5 in the innate response to RV infection in primary HBECs, the target cell for RV infection within the lower airway *in vivo*. We found that HBECs did not respond to the ligand R-848. Importantly, TLR3, and RNA helicase mediated signaling was required for maximal IFN-β, IFN-λ and pro-inflammatory cytokine gene expression, showing that RIG-I is required for anti-viral defense against Picornaviruses. Furthermore, RIG-I and MDA5 were virus inducible genes, induced early via a TLR3/TRIF pathway, indicating that TLR3 acts as an initial endosomal sensor and must induce the RNA helicases for maximal anti-viral defense during the course of infection. Thus the innate response to RV infection requires co-ordinated endosomal, and cytoplasmic recognition pathways, both of which contribute to IFN and cytokine production.

## Results

### Primary HBECs express TLR3, and RNA helicases RIG-I and MDA5, which are RV inducible genes with similar expression kinetics to RV induced IFN-β and IFN-λ

**Figure 1:**
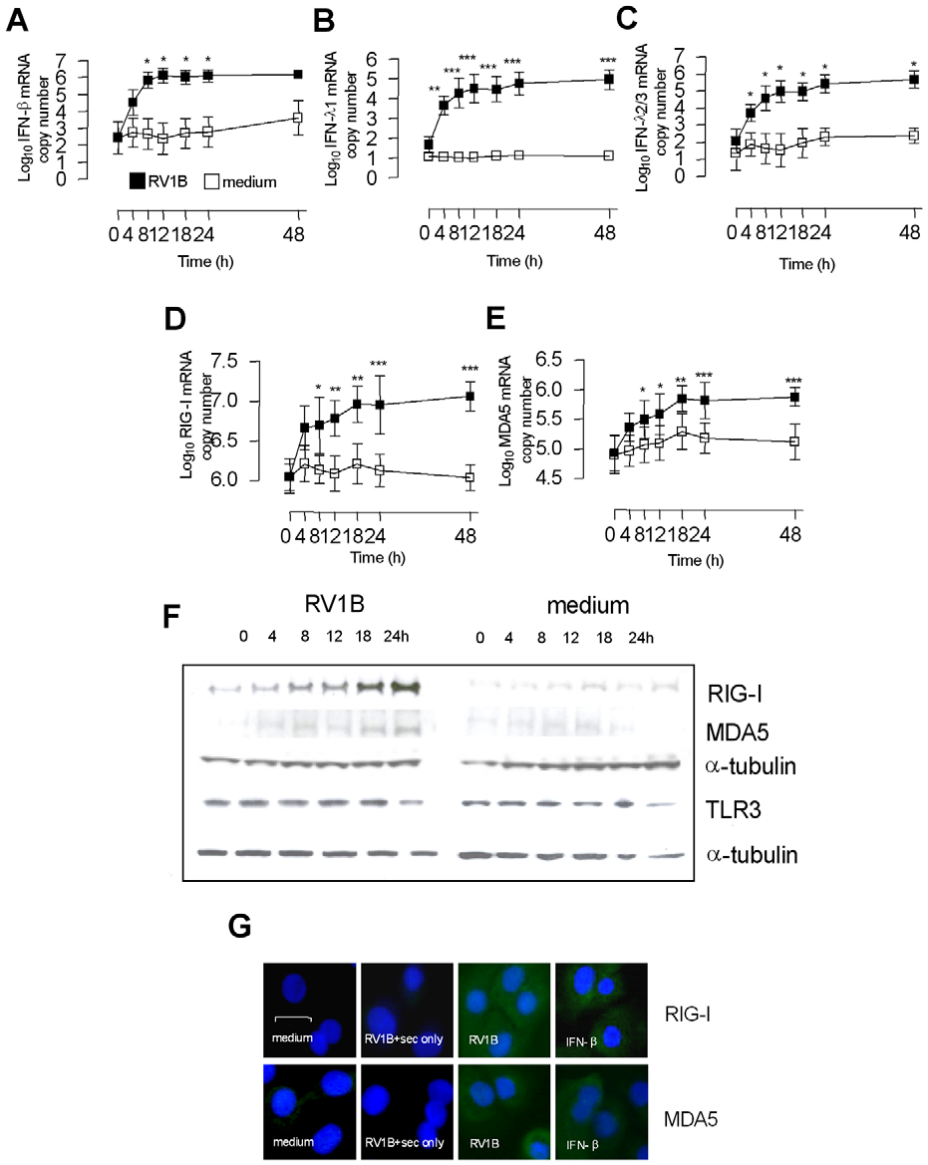
Kinetics of RV1B induced IFN-β, IFN-λs, RIG-I and MDA5 mRNA and protein expression in HBECs. HBECs were infected with RV1B or treated with medium and RNA and protein analysed over time. RV1B induced IFN-β (A), IFN-λ1 (B) and IFN-λ2/3 mRNA (C) in a time dependent manner, visible by 4 h, and peaking at 12–48 h. RV1B infection also induced RIG-I (D) and MDA5 mRNA (E), in a time dependent manner, peaking at 18–48 h. RV1B infection also induced RIG-I and MDA5 protein (F) in a similar time dependent manner, visible by 8 h post infection by western blotting. Medium treated cells exhibited little or no RIG-I or MDA5 protein during the timecourse. TLR3 protein levels were present in medium treated cells, and did not change during the course of RV1B infection. Immunofluorescence identified both cytoplasmic RIG-I and MDA5 to be induced after RV1B or IFN-β treatment at 24 h, compared to medium treated cells, or cells stained with secondary antibody only. Horizontal line indicates 20 µm scale. Staining for both helicases was observed within the cytoplasm (in green, G). *p<0.05, **p<0.01, ***p<0.001, RV1B infected versus medium treated cells. mRNA data was generated from 6 independent experiments utilizing 3 independent HBEC donors, 2 experiments per donor.

We first sought to assess the relationship between TLR3/7/8, and the RNA helicases in RV infection in bronchial epithelial cells. Initial studies showed that HBECs encoded mRNA and protein for TLR3, but did not induce IFN-β, IFN-λ, RIG-I or MDA5 mRNA in response to the TLR7/8 ligand R848 (Table S1 in Supporting Information S1), consistent with other studies showing that lung bronchial epithelial, alveolar and airway smooth muscle cells do not respond to TLR7/8 agonists [9], [37], [38]. The presence and/or absence and virus induction of TLR3 and RNA helicases was then investigated. Time course analysis in HBECs demonstrated that IFN-β was induced by 8 h post infection, and 4 h for IFN-λs post RV1B infection. IFN-β peaked at 12 h and remained at high levels until 48 h, while the IFN-λs remained elevated from 12 h and peaked at 48 h (Figure 1A–C). In the same experiments, RIG-I and MDA5 mRNA levels were also measured, and were induced by RV1B by 8 h, peaked by 18 h post infection and remained at high level until 48 h post infection (Figure 1D–E). At the protein level, RIG-I and MDA5 protein were both observed by 8 h following RV1B infection, and showed maximal levels at 18–24 h (Figure 1F). Uninfected cells and cells sampled at time 0 h exhibited almost non-detectable expression of RIG-I and MDA5 protein, suggesting that in the absence of active infection, RIG-I and MDA5 proteins are absent or expressed at very low level. TLR3 protein however was present in both infected and uninfected cells, and the levels did not change over the time course of RV1B infection. Similar data was observed for TLR3 mRNA (data not shown). The cytoplasmic staining of RIG-I and MDA5 were confirmed using immunofluorescence, with RV1B and IFN-β inducing both RIG-I (Figure 1G *upper panel*) and MDA5 (Figure 1G *lower panel*) at 24 h post treatment in HBECs, compared to cells treated with medium, or RV1B infected cells stained with a secondary antibody only.

### RIG-I and MDA5 are induced in columnar bronchial epithelial cells upon experimental RV challenge *in vivo*

**Figure 2:**
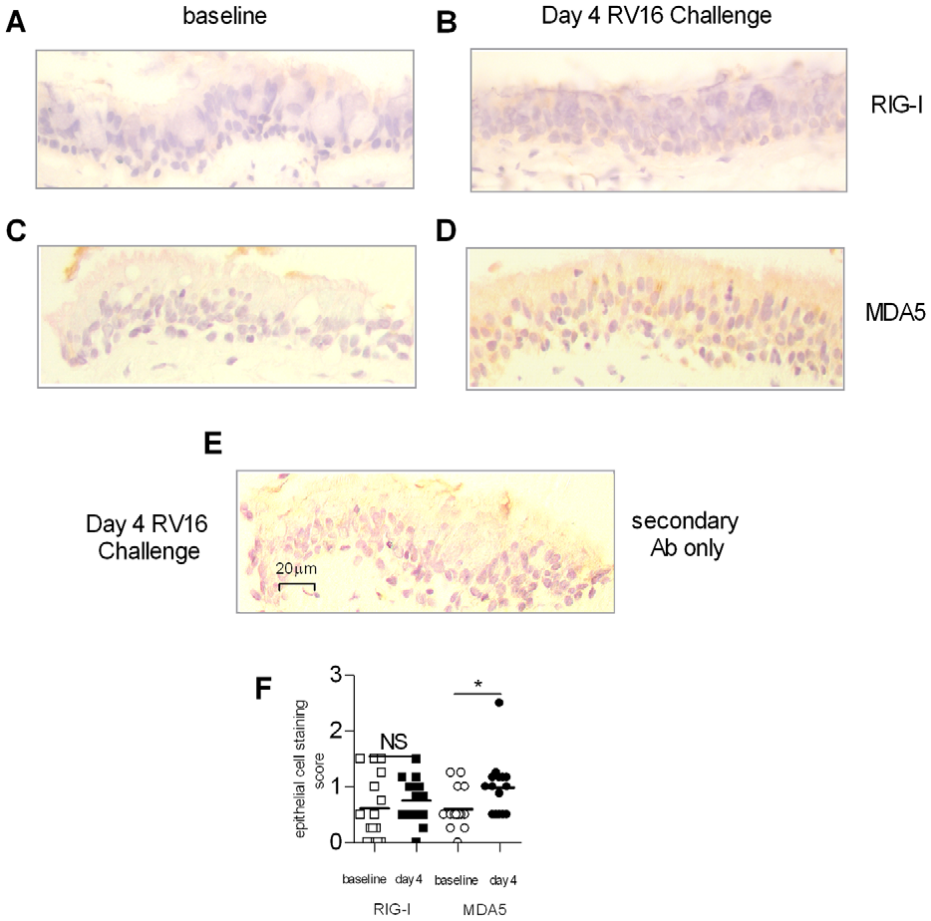
RIG-I and MDA5 expression in bronchial biopsy tissue following *in vivo* RV16 infection. Normal individuals underwent fibre-optic bronchoscopy at baseline or day 4 following RV16 challenge, and bronchial biopsies were taken, and assessed for RIG-I protein by immunohistochemistry at baseline (A) and day 4 post RV16 challenge (B). Biopsies were also assessed for MDA5 protein at baseline (C) and day 4 post RV16 challenge (D). Positive staining is indicated by the brown coloration, localized to the columnar epithelial cells. Background staining was assessed by incubating a Day 4 biopsy with secondary antibody only (E). Staining scores of bronchial epithelium showing increases at day 4 compared to baseline (F) *p<0.05 as indicated, NS  =  not significant. Scale is indicated by the horizontal bar.

In order assess the baseline expression and the RV mediated induction of RIG-I and MDA5 protein in bronchial epithelium *in vivo*, bronchial biopsies were taken from 15 normal adult volunteers before experimental RV16 infection (baseline) or at day 4 post infection and stained for RIG-I and MDA5 by immunohistochemistry. Representative staining of biopsy samples are presented in Figure 2A–E. The degree of epithelial staining for RIG-I and MDA5 protein were scored quantitatively, and presented in Figure 2F. RIG-I (A) and MDA5 (C) had little staining at baseline, and MDA5 was increased at day 4 post RV16 infection, (Figure 2D). Scoring of columnar epithelial staining (F) showed that MDA5 at day 4 was significantly higher than baseline (*p*<0.05), however RIG-I levels were not significantly different at day 4 versus baseline (Figure 2B).

### TLR3, RIG-I and MDA5 are required for maximal RV induced IFN-β, and IFN-λ

**Figure 3:**
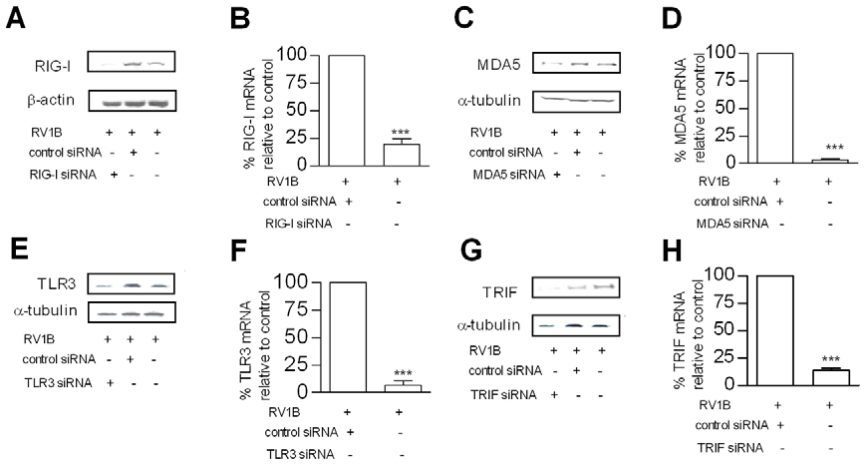
Effects of each siRNA on RIG-I, MDA5, TLR3 and TRIF mRNA and protein levels. HBECs were transfected with siRNA specific for RIG-I or control siRNA, infected with RV1B and RIG-I protein (A) and mRNA (B) measured. HBECs were transfected with MDA5 specific siRNA or control siRNA and infected with RV1B and MDA5 protein (C) and mRNA (D) measured. HBECs were transfected with siRNA specific for TLR3 or control siRNA and infected with RV1B and TLR3 protein (E) and mRNA (F) measured. HBECs were transfected with siRNA specific for TRIF or control siRNA and infected with RV1B and TRIF protein (G) and mRNA (H) measured. *p<0.05, **p<0.01, ***p<0.001 versus control siRNA or as indicated. mRNA data was generated from 6 independent experiments utilizing 3 independent HBEC donors, 2 experiments per donor.

**Figure 4 ppat-1001178-g004:**
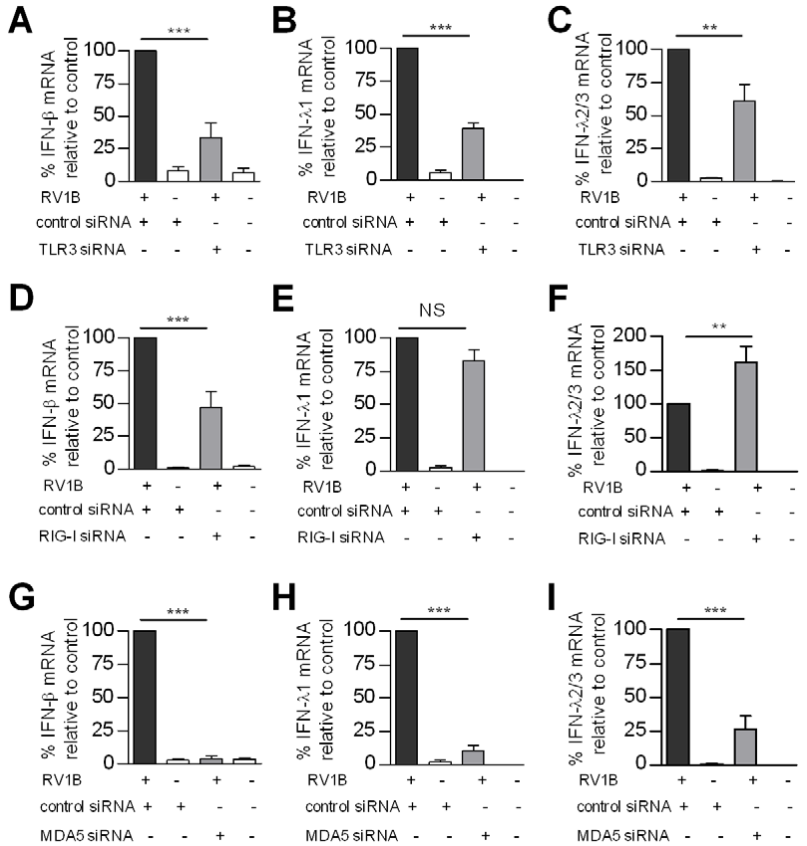
Role of TLR3, RIG-I and MDA5 in RV1B induced IFN-β, and IFN-λ gene expression in HBECs. HBECs were transfected with control siRNA or siRNA specific for TLR3 (A–C), RIG-I (D–F), MDA5 (G–I), and infected with RV1B. IFN-β (A,D,G), IFN-λ1(B,E,H) and IFN-λ2/3 (C,F,I) mRNA were measured 24 h post infection **p<0.01, ***p<0.001 versus control siRNA, NS = not significant. mRNA data was generated from 6 independent experiments utilizing 3 independent HBEC donors, 2 experiments per donor.

The PRRs involved in RV infection and induction of innate responses are largely unknown. In order to assess the role of TLR3, RIG-I and MDA5 in RV induced IFNs we used RNA interference with specific small interfering RNA (siRNA) to knockdown each PRR in HBECs *in vitro*, prior to RV infection. Initial experiments demonstrated that siRNA generated >75% knockdown of target mRNA at 24 h post treatment, and this knockdown was evident until 48 h post treatment (data not shown). Therefore, siRNA was delivered 24 h before infection, and total RNA harvested 24 h post RV1B infection, giving a total siRNA transfection time of 48 h. The knockdown of each target mRNA was confirmed in each experiment, and knockdown of target protein also confirmed at 48 h post transfection (Figure 3A–H). Also, experiments were performed in the absence of RV infection to examine the effects of siRNA on endogenous IFN and pro-inflammatory cytokine gene expression. Each siRNA did not significantly induce any of the IFNs or pro-inflammatory cytokines studied (Table S2 & S3 in Supporting Information S1). Furthermore, RIG-I and MDA5 siRNA were highly specific, RIG-I siRNA did not affect endogenous MDA5 gene expression, and MDA5 siRNA did not affect endogenous RIG-I gene expression (data not shown). TLR3, RIG-I and MDA5 siRNA all reduced RV1B induced IFN-β compared to control siRNA (Figure 4A,D,G respectively). In contrast, siRNA targeting RIG-I did not reduce IFN-λ1 mRNA, (Figure 4E) however siRNA specific for TLR3 and MDA5 reduced IFN-λ1 (Figure 4B,H respectively). RIG-I siRNA enhanced RV induced IFN-λ2/3 mRNA (Figure 4F), while both MDA5 and TLR3 reduced RV1B induced IFN-λ2/3 (Figure 4C,I). These data suggest that TLR3, RIG-I and MDA5 are all required for IFN-β, and TLR3 and MDA5 for IFN-λ, however the importance of RIG-I in IFN-λs is less clear. To confirm these findings, we next used siRNA specific for the TLR3 adaptor TRIF, and the RNA helicase adaptor Cardif. We found that RV1B induced IFN-β and IFN-λ1 mRNA expression was inhibited by both siRNA to TRIF and Cardif, while IFN-λ2/3 was inhibited by TRIF siRNA only compared to control siRNA (Figure S1).

### TLR3, RIG-I and MDA5 are required for maximal RV induced pro-inflammatory cytokine gene expression

**Figure 5 ppat-1001178-g005:**
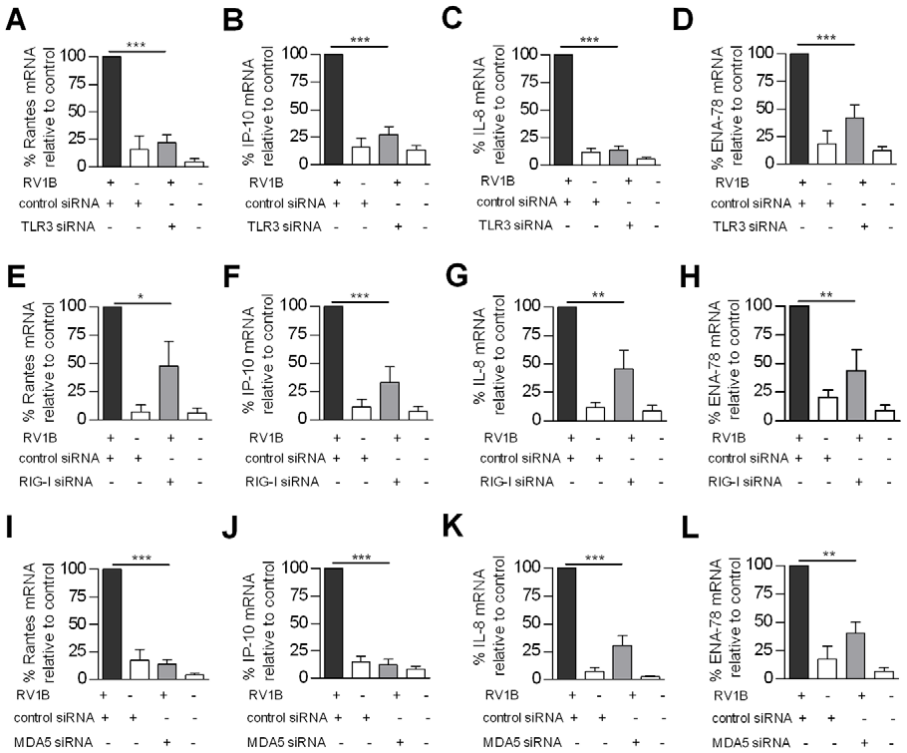
Role of TLR3, RIG-I and MDA5 in RV1B induced pro-inflammatory cytokine gene expression in HBECs. HBECs were transfected with control siRNA or siRNA specific for TLR3 (A–D), RIG-I (E–H), MDA5 (I–L), and infected with RV1B. Rantes (A,E,I), IP-10 (B,F,J), IL-8 (C,G,K), and ENA-78 (D,H,L) mRNA were measured 24 h post infection *p<0.05, **p<0.01, ***p<0.001 versus control siRNA. *p<0.05, **p<0.01, ***p<0.001 versus control siRNA or as indicated. mRNA data was generated from 6 independent experiments utilizing 3 independent HBEC donors, 2 experiments per donor.

The role of TLR3, RIG-I and MDA5 on the T cell chemokines rantes and IP-10, and the neutrophil chemokines IL-8 and ENA-78 were also investigated. Figure 5 shows that TLR3, RIG-I and MDA5 siRNA all reduced RV1B induced rantes compared to control siRNA (Figure 5A,E,I). Likewise, TLR3, RIG-I and MDA5 siRNA all reduced RV induced IP-10 (Figure 5B,F,J). TLR3, RIG-1 and MDA5 siRNA also reduced RV induced IL-8 mRNA (Figure 5C,G,K). Finally TLR3, RIG-I and MDA5 siRNA also reduced RV1B induced ENA-78 compared to control siRNA (Figure 5D,H,L). In support of the above, siRNA specific for Cardif and TRIF reduced all RV1B induced pro-inflammatory cytokines compared to control siRNA (Figure S2).

### Inhibition of RIG-I and MDA5 increased RV replication

**Figure 6 ppat-1001178-g006:**
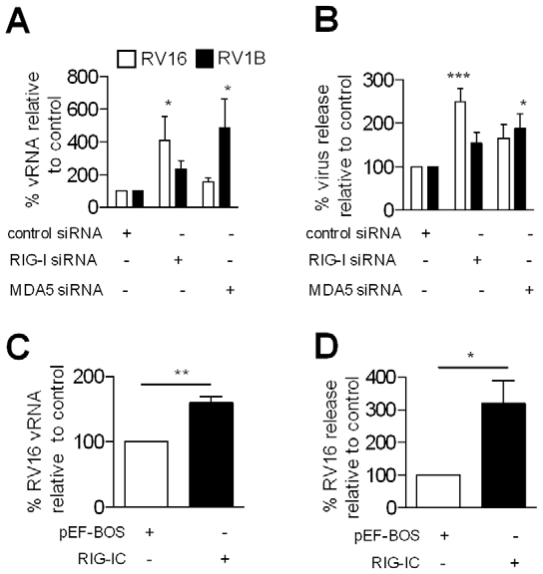
Effects of RIG-I and MDA5 abrogation on RV replication in HBECs. HBECs were transfected with siRNA specific for RIG-I, MDA5 or control siRNA, infected with RV1B or RV16 and vRNA determined by PCR at 24 h (A) or RV release by titration at 48 h (B). BEAS-2B cells were transfected with 0.5 µg RIG-IC or pEF-BOS control, and RV16 vRNA (C) and virus release (D) determined. mRNA data was generated from 5 independent experiments utilizing 3 independent HBEC donors, 2 experiments per donors 1,2 and a one experiment for donor 3; and 4 independent experiments for BEAS-2B cells.

As RIG-I and MDA5 were required for RV1B induced IFN-β and both IFN-β and IFN-λ gene expression respectively, we next reasoned that their role was not redundant and that abrogation of either RNA helicase would result in increased RV replication in HBECs. Figure 6A demonstrates that after 24 h post infection, RV16 RNA levels were increased after transfection with siRNA specific for RIG-I compared to control siRNA. RV1B RNA levels were also increased. Also, using siRNA specific for MDA5, an increase in RV1B RNA was observed, compared to cells transfected with control siRNA, and RV16 RNA levels were slightly increased compared to control siRNA. In the same experiments, virus release was also determined by titration assay, 48 h after infection. Figure 6B demonstrates that transfection with RIG-I specific siRNA resulted in increased RV16 release compared to control siRNA and slightly increased RV1B virus release. Conversely, transfection with MDA5 specific siRNA resulted in higher RV1B release, and a small increase in RV16 virus release compared to control siRNA. Furthermore, transfection of the bronchial epithelial cell line BEAS-2B, with dominant negative RIG-I (RIG-IC), resulted in enhanced replication of RV16 RNA (Figure 6C), at 24 h and RV16 virus release at 48 h post infection (Figure 6D). Transfection of constitutively active RIG-I (ΔRIG-I) resulted in up to 60 fold suppression of RV1B and 47 fold suppression of RV16 virus release at 48 h (Figure S3). This data further implicates the RNA helicase RIG-I in RV recognition and induction of anti-viral activity.

### RV induced RIG-I and MDA5 gene expression involved TLR3/TRIF signalling

**Figure 7:**
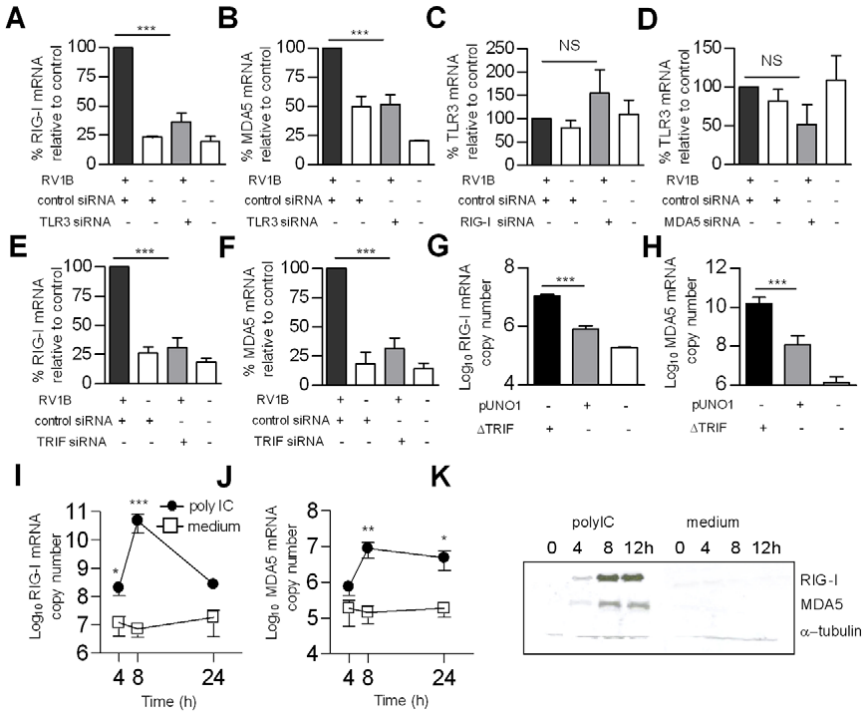
Role of TLR3 and TRIF in RV1B induced RIG-I and MDA5 expression. HBECs were transfected with siRNA specific for TLR3 or control siRNA (A,B) RIG-I or control siRNA (C) or MDA5 or control siRNA (D), TRIF or control siRNA (E,F) infected with RV1B, and RIG-1 (A,E), MDA5 (B,F) or TLR3 mRNA (C,D) measured at 24 h. HBECs were transfected with ΔTRIF or pUNO1 plasmids, or were left untransfected, and RIG-I (G) and MDA5 mRNA (H) measured 24 h post transfection. HBECs were treated with the TLR3 ligand polyIC or medium and RIG-I (I) and MDA5 mRNA (J) measured at various time points, or RIG-I and MDA5 protein assessed by western blotting over time (K). *p<0.05, **p<0.01, ***p<0.001 versus control siRNA, pUNO1, or medium treated cells, NS = not significant. mRNA data was generated from 5 independent experiments utilizing 3 independent HBEC donors, 2 experiments per donors 1,2 one experiment for donor 3.

**Figure 8:**
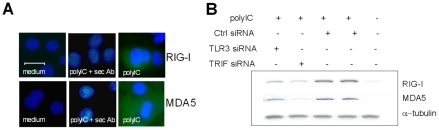
Role of TLR3 and TRIF in polyIC induced RIG-I and MDA5 protein. HBECs were treated with polyIC or medium and examined using immunofluorescence at 24 h for RIG-I *(upper panel,* A*)* and MDA5 *(lower panel,* A*)*. Both cytoplasmic RIG-I and MDA5 (in green) was induced after polyIC treatment at 24 h, compared to medium treated cells, or cells stained with secondary antibody only. Horizontal line indicates 20 µm scale. HBECs were treated with polyIC or medium for 8 h and the effects of TLR3 and TRIF siRNA on polyIC induced RIG-I and MDA5 assessed by western blotting (B). TLR3 and TRIF siRNA reduced RIG-I and MDA5 protein compared to control siRNA. α-tubulin was used to control for protein loading.

As RV1B infection induced both early RIG-I and early MDA5 protein and mRNA production, and both are required for maximal anti-rhinoviral activity, we sought to identify the receptor(s) responsible for RNA helicase induction. As RV enters via the endosome, we hypothesized that TLR3 was responsible for increased RIG-I and MDA5 gene expression. In order to investigate the relationship between TLR3/TRIF signaling and RIG-I and MDA5 gene expression in RV infection, we next assessed if specific knockdown of TLR3 and the adaptor TRIF affected RV1B induced RNA helicase gene expression. Figure 7 demonstrates that siRNA specific for TLR3 reduced RV1B induced RIG-I mRNA versus control siRNA, (Figure 7A), and reduced RV1B induced MDA5 mRNA (Figure 7B). Consistent with the sequential affect of TLR3 on RNA helicase induction, we found that RIG-I specific siRNA did not affect TLR3 mRNA levels compared to control siRNA (Figure 7C), and MDA5 specific siRNA also did not affect TLR3 mRNA levels compared to control siRNA (Figure 7D). Furthermore, siRNA specific for the TLR3 adaptor TRIF, reduced RV1B induced RIG-I mRNA (Figure 7E) and also RV1B induced MDA5 mRNA compared to control siRNA (Figure 7F). In each experiment, knockdown of TRIF mRNA by TRIF specific siRNA was confirmed, and we also confirmed knockdown of TRIF protein at 48 h post transfection (Figure 3G,H). Using a plasmid encoding constitutively active TRIF (ΔTRIF), the reverse of the above results were obtained in HBECs (Figure 7G,H). Transfection with ΔTRIF significantly increased both RIG-I and MDA5 gene expression, compared to empty vector control (pUNO1). Furthermore, incubation of HBECs with the TLR3 ligand polyIC induced RIG-I mRNA from 4–24 h post treatment (Figure 7I) and MDA5 from 8–24 h (Figure 7J). PolyIC treatment also induced RIG-I and MDA5 protein by 4–12 h post treatment as shown by western blotting (Figure 7K) and immunofluorescence (Figure 8A). Finally siRNA specific for TLR3 or TRIF reduced polyIC induced RIG-I and MDA5 protein (Figure 8B), strongly implicating TLR3/TRIF mediated signal transduction in RV induced RNA helicase induction in HBECs.

### RV1B infection *in vivo* resulted in initial increased RIG-I and MDA5 expression in the absence of type I and type III IFN signalling

**Figure 9:**
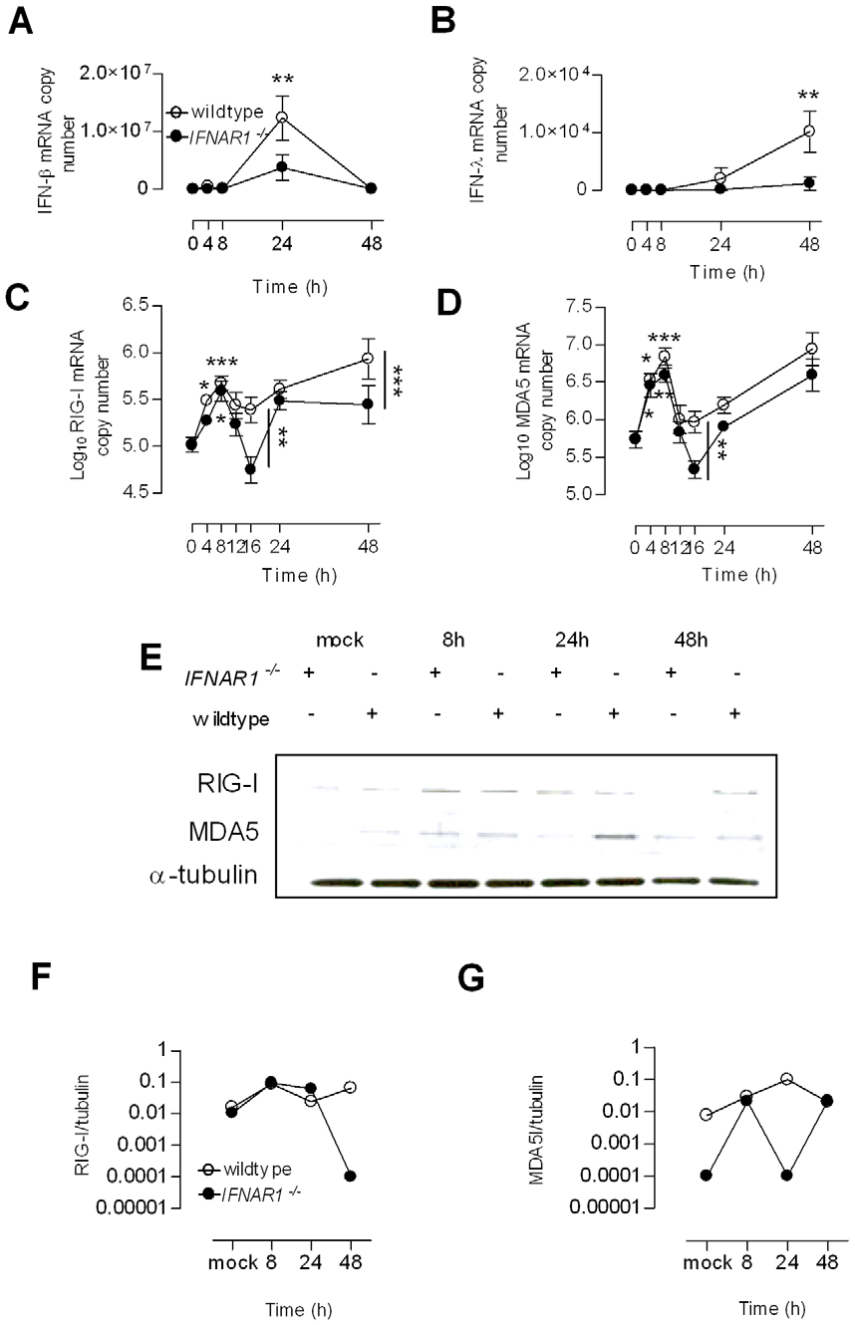
Expression of RIG-I and MDA5 mRNA and protein in wildtype and *IFNAR1* deficient mice. Wildtype 129/SvJ and *IFNAR1^−/−^* mice were infected with RV1B and lung protein and mRNA analysed over time. In wildtype mice, and to a lesser extent, *IFNAR1^−/−^* mice, RV1B induced IFN-β mRNA (A), and IFN-λ mRNA only in wildtype mice (B), and induced RIG-I in both wildtype and *IFNAR1^−/−^* mice (C). RV1B also induced MDA5 in both wildtype and *IFNAR1^−/−^* mice (D). In both wildtype and *IFNAR1^−/−^* mice, RIG-I and MDA5 mRNA induction was evident by 8 h post infection. RV1B also induced RIG-I and MDA5 protein by 8 h in both wildtype and *IFNAR1^−/−^* mice, measured by western blot (E), and RIG-I and MDA5 protein as a ratio of α-tubulin protein measured by densitometry over time is also shown (F&G respectively) *p<0.05, **p<0.01 ***p<0.001 versus time 0 h or as indicated.

As RV induced RIG-I and MDA5 occurred early, and in a TLR3/TRIF dependent manner, and as the TLR3 and TRIF pathway leads to IRF3 activation and IFN-β and IFN-λ induction, we assessed the role of IFN signalling in RV1B induced RIG-I and MDA5. In our *in vitro* experiments with HBECs, it was difficult to rule out the role of endogenous, or RV induced early IFN-β/λ inducing RIG-I and MDA5. We therefore ultilised *IFNAR1* deficient mice, in a mouse model of RV1B infection [39]. *IFNAR1* deficient mice are devoid of IFN-α/β signaling, and spleen cells have recently been shown to have reduced IFN-λ (IL-28B) production *in vitro* and lack IFN-λ within the vagina *in vivo* [40]. Figure 9 shows that following RV1B infection, *IFNAR1* deficient mice produced a low level of IFN-β within the lung but did not produce IFN-λ, however wildtype controls produce both IFN-β mRNA at 24 h (Figure 9A) and IFN-λ mRNA at 48 h (Figure 9B). At 8 h post infection, RV1B infection of *IFNAR1* deficient mice and wildtype mice both resulted in increased RIG-I (Figure 9C) and MDA5 gene expression compared to time 0 h, (Figure 9D). The induction of RIG-I and MDA5 was biphasic for both *IFNAR1* deficient and wildtype mice, wildtype mice produced higher levels of RIG-I and MDA5 mRNA at 16 h post infection, and at 48 h post infection for RIG-I only. Cytoplasmic protein from lung homogenates were extracted and probed for RIG-I and MDA5 protein by western blotting. Both *IFNAR1^−/−^* and wildtype mice exhibited increased RIG-I and MDA5 protein at 8 h compared to mock infected controls. At later time points (24 h and 48 h) wildtype mice had more RIG-I and MDA5 protein compared to *IFNAR1* deficient mice, likely caused by IFN signaling (blots are shown in Figure 9E and densitometry compared to α-tubulin shown in Figure 9F&G). This data therefore shows that initial RV induced RIG-I and MDA5 is IFN independent, and that at later time points, further virus replication increases in RIG-I and MDA5 gene expression later on in *IFNAR1^−/−^* mice, and in wildtypes further virus replication and IFN signaling events contribute to later lung RNA helicase mRNA expression.

## Discussion

Viral dsRNA and ssRNA are recognised by at least two independent pattern recognition pathways, composed of TLR3, and TLR7/8 in the endosome and the RNA helicases RIG-I, MDA5 in the cytoplasm. Previous studies have employed well established models of virus infection, however similar studies using viruses important to human disease in their natural host cell type *in vivo* are largely yet to be performed. In the present study, we describe the role of TLR3 and RNA helicases in the recognition of RV infection in primary HBECs, the main cell type infected in the lower respiratory tract *in vivo*. RV is an important human pathogen, responsible for a range of illnesses including asthma exacerbations. We have used both major group RV16 and minor group RV1B (approximately 77% genome identity) in our studies, as both these groups represent the majority of RVs involved in human disease. Understanding the basis of RV recognition and signalling leading to IFN and pro-inflammatory cytokines could potentially lead to new therapeutic targets for RV associated illnesses.

We found that TLR3, RIG-I and MDA5 were required for IFN-β while MDA5 and TLR3 were required for maximal IFN-λ1 and IFN-λ2/3 mRNA expression. We then hypothesized that the most likely explanation for the dual requirement of both endosomal and cytoplasmic recognition systems and the importance of both RIG-I and MDA5 for IFN-β expression was that the endosomal and cytoplasmic recognition pathways were in some way linked, and their sequential activation was required for maximal IFN-β and IFN-λ gene expression. This is in contrast to other studies, which have observed mostly cell type specific differences concerning RIG-I/MDA5 and the TLR family members, with murine embryonic fibroblasts (MEFs) and conventional dendtitic cells (DCs) utilizing RIG-I and MDA5 for virus induced IFN production [25], [36] while plasmacytoid DCs use TLRs including TLR3 [35], [36]. Despite these findings, a recent study has reported the induction of type I IFN after i.p. injection of polyIC, to be reduced in *TRIF^−/−^* and *IPS-1^−/−^* double deficient mice, compared to either *IPS^−/−^* or *TRIF^−/−^* mice [41]. A number of recent *in vitro* studies have also reported a role for both TLR and RNA helicase signalling in polyIC induced responses, [42]–[45]. In short, explanations for differences between these results are likely due to cell type dependence on one pattern recognition system versus another, and the models of virus infection employed. We argue that for structural cells, including epithelial cells at mucosal surfaces, these cells may have evolved a dual dependency on both endosomal and intracellular recognition systems, which may be dissimilar for leukocytes including DCs. Hence as a first line defense against viral infection, efficient IFN production is an outcome of both TLR and RNA helicase mediated signalling working together.

The siRNA experiments we performed have demonstrated the requirement of TLR3 and MDA5 for IFN-λs, but TLR3, RIG-I and MDA5 for IFN-β gene expression. TLR3, RIG-I and MDA5 were also all important for a range of pro-inflammatory cytokines, including T cell chemokines rantes and IP-10 and neutrophil chemokines IL-8 and ENA-78. These results confirm and expand on recent data by Wang et al [46], using BEAS-2B cells and specific siRNA transfection, Wang et al showed that MDA5, TRIF but not RIG-I was important for RV induced IFN-β, IFN-λs and several interferon stimulated genes (ISGs). The role of MDA5 was confirmed in primary tracheobronchial epithelial cells. However why or how MDA5 and TLR3 are both required for RV infection in these studies was not analyzed in any depth. Our observation of RIG-I being important for IFN-β in HBECs could be due differences between BEAS-2B cells and HBECs, or differences in efficiency of siRNA knockdown of target mRNA. Our data also show that the RIG-IC DN also increases RV replication in BEAS-2B cells, again suggests a role for RIG-I in RV responses. Why RIG-I was not required for IFN-λ expression in our studies is unclear. Possible explanations for these results could be that MDA5 siRNA was more consistent at reducing the target mRNA compared to RIG-I siRNA, or that at the time point studied (24 h), MDA5 is more important than RIG-I in IFN-λ expression. IFN-β and the IFN-λs are both IRF3 responsive [47], and it is possible that for RV infection, MDA5 is more efficiently activated, resulting in robust signalling and IRF3 activation. Recently, it has been shown that IRF3 activation is complex, requiring multiple kinases for maximal phosphorylation and activation [48]. It is possible that differences exist between RIG-I and MDA5 signaling to IRF3, or IFN-β and IFN-λ promoters have different requirements for IRF3 activation. It has also previously been observed that siRNA transfection can interfere with endogenous RNA sensing molecules [49], [50] and induce spontaneous IFN or cytokine production. All our siRNA were used to minimize potential off-target effects; they were used as pools of four individual siRNAs, and were designed with minimal known stimulatory sequences and also contained 3′ UU overhangs to minimize activation of RIG-I, which can be activated by siRNA [49]. We were careful to assess the likelihood of spontaneous induction of IFN or cytokines studied by all siRNA, and we did not observe significant induction for control or any specific siRNA. Therefore, we are confident that our results using siRNA are accurately describing the role of each molecule in RV dependent responses, and are not confounded due to siRNA recognition by endogenous processes or are the result of obvious off-target effects.

Initial experiments into the specificity of RIG-I suggested it bound *in vitro* transcribed and/or 5′-triphosphorylated ssRNA [33]–[35], and therefore could not recognize the RNA of picornaviruses such as encephalomyocarditis virus (EMCV) which do not synthesize 5′-triphosphosphorylated ssRNA. This has been questioned recently with the observation that RIG-I can bind low weight dsRNA [32]. Other than EMCV, little is known about the role of RIG-I in the infection of Picornaviruses. Our data provide definitive evidence that both RIG-I and MDA5 are important in innate responses to RV infection. Knockdown of both RIG-I and MDA5 produced higher viral loads of major group RV16 and minor group RV1B, again providing evidence that RIG-I can recognize the RNA of Picornaviruses. As the 7kB RV genome replicates in a RNA dependent manner, we argue that dsRNA molecules are present during replication in the cytoplasm. Hence, it is plausible that dsRNA of differing sizes could potentially ligate both RNA helicases. Future studies to confirm these interactions, such as immunoprecipitation studies are necessary to investigate the exact nature of the RNA species that RIG-I and MDA5 are binding to in the context of RV infection in HBECs.

Having established that TLR3, RIG-I and MDA5 were all required for innate responses and that RIG-I and MDA5 protein was not constitutively expressed in HBECs, whereas TLR3 was constitutively expressed, we hypothesized that RIG-I and MDA5 could be induced by TLR3 activation. While we did not study the early events of viral entry, RV has been used as a model Picornavirus and their biology has been extensively studied. RV enters via the endosome where the acidified environment is essential to viral uncoating and release of +ve sense ssRNA. [51], [52]. Despite being a ssRNA virus, the 7kB ssRNA genome contains some secondary structures [53], [54], including the 5′ multiple stem loop structure, containing the ribosome entry site, which has been previously visualized in endosomes during virus uncoating [55]. As RV infection requires the formation of mature endosomes, and contains dsRNA structures, thus our results could be explained by TLR3 sensing these events during viral entry; and initiate signaling leading to IFN, and RIG-I and MDA5 gene expression during the first few hours of infection. Further experiments are required to prove or disprove this idea however. HBECs were unresponsive to the TLR7/8 ligand R848, strongly suggesting this cell type lacks these TLRs, and that TLR3 rather than TLR7/8 is involved in RIG-I and MDA5 induction. TLR3 has previously been implicated in RV recognition in BEAS-2B cells [56] and HBECs [9]. Interestingly, unlike BEAS-2B cells, we found that TLR3 was not virus inducible in undifferentiated HBECs. In both models however, TLR3 is constitutively expressed, enforcing the hypothesis that TLR3 is an initial sensor of viral nucleic acid in this cell type.

A range of experiments in HBECs demonstrated that RIG-I/MDA5 induction was TLR3/TRIF dependent. Very recently, co-operation between RIG-I/MDA5 and TLR3 has been suggested in a murine model of coxsackievirus infection [57]. TLR3 was absolutely required for defense against coxsackievirus infection, by inducing IFN-γ. The authors suggest that TLR3 mediated induction by IFN-γ may work in parallel with RIG-I/MDA5 inducing type I IFN, and further suggested that these responses may be coupled, although potential mechanisms for this were not explored. We believe however that our study is the first to provide definitive evidence of endosomal and intracellular PRRs working in concert. We argue that as RV enters via endosomes, yet most of the dsRNA load occurs in the cytoplasm, the idea of two related pattern recognition systems seem plausible. RV replicates in the cytoplasm, and produces multiple centers of RNA dependent RNA replication, therefore increases in the RNA helicases can be viewed as a mechanism to continually monitor the intracellular RNA load (likely dsRNA and ssRNA), and through RNA helicases, induce IFN and cytokines consistently, during the course of infection. The upregulation of IFN-β, IFN-λ and T cell and neutrophil cytokines are highly important for the control of virus replication and acute inflammatory responses within the airway (for a summary, see Figure S4). It would be extremely interesting to study other common human respiratory viruses which preferentially infect bronchial epithelial cells, or viruses that infect other mucosal surfaces such as the gut, to investigate if maximal IFN and cytokine responses require both TLR3, and RIG-I/MDA5.

Finally, we assessed the role of IFN-α/β signaling in RIG-I and MDA5 induction. RIG-I and MDA5 are ISGs, a consistent observation in many different cell types and models [24], [57]–[60]. We have also observed that in HBECs, RIG-I and MDA5 are IFN-β and IFN-λ inducible (data not shown). As RIG-I and MDA5 are IFN inducible, our in vitro experiments could not rule out the possibility of the effects of low level IFN inducing RIG-I and MDA5. Our data *in vivo*, however clearly show that RIG-I and MDA5 can be induced in the absence of IFN-β and IFN-λ, *IFNAR1^−/−^* mice, which cannot respond to type I IFN and are unable to produce IFN-λ in the lung, still produced RIG-I and MDA5 mRNA and protein upon RV1B infection. Also, in HBECs with intact IFN responses, RIG-I/MDA5 mRNA was upregulated early, by 4–8 h, and we argue this quick response is likely IFN independent.

We believe this is the first report of sequential involvement or collaboration of TLR and RNA helicase mediated pathways for innate defense against a virus infection. Our overall model is depicted in Figure S4. The model argues that RIG-I and MDA5 that are not well expressed in uninfected cells are both virus inducible via the constitutively expressed TLR3/TRIF, and later IFN inducible. Upon RV infection, TLR3 signaling in the endosome gives quick induction of new RIG-I and MDA5. Importantly, the model highlights the need for RNA helicase induction to be quick, in a few hours in infected cells. After several hours, as virus moves out of the endosome and into the cytoplasm, newly synthesized virus RNA is sensed by RIG-I/MDA5. This is where the majority of viral nucleic acid will be for the rest of the infection cycle, and is likely key in the induction of innate immune response to RV infection. At later time points, the actions of IFN-β and IFN-λs may further induce RIG-I and MDA5 in infected and non-infected cells. In non-infected cells, the presence of IFN may “warn” neighboring cells about the presence of viruses, and prepare the epithelium through upregulation of interferon inducible genes including RIG-I and MDA5.

Our initial interest in PRRs important in RV infection and IFN expression came from studies in asthmatic individuals which showed bronchial epithelial cells from asthmatics had very low levels of IFN-β and IFN-λ expression and increased RV replication compared to non-asthmatic controls [17], [18]. It is currently believed that IFN-β, or IFN-λs could contribute to the outcome of asthma exacerbations. While these studies implicate the bronchial epithelium as a key producer of IFN-β and IFN-λs in RV infections, it is unclear which IFN is more important in protection. Understanding the regulation of both type I and type III IFNs is therefore a research goal for identifying why asthmatics have deficient innate responses to RV infection. The results of the present study have identified the importance of RIG-I, MDA5 and TLR3 in RV induced IFN, therefore future studies should scrutinize these pathways in asthmatics and non-asthmatics to ascertain if asthmatics have defective signaling leading to decreased IFN production.

In summary we provide evidence that the dsRNA receptor TLR3 acts to induce both RIG-I and MDA5 gene and protein expression in HBECs in a model of RV infection. Both RIG-I and MDA5 were required for maximal IFN and pro-inflammatory cytokine induction, and control of RV replication indicating that they have non-redundant roles in RV infection. The data support a model that in HBECs, TLR3 but not TLR7/8 operate in concert with RIG-I/MDA5, and are together required for innate responses to RV infection. As asthmatic HBECs have reduced RV inducible IFN-β and IFN-λ expression compared to non-asthmatic cells, both the TLR3 and RNA helicase pathways warrant further exploration in order to ascertain why these cells produce reduced IFN expression.

## Materials and Methods

### Cells and viruses

HBECs from non-asthmatic, non-smoking individuals were obtained from a commercial source (Clonetics, Wokingham, UK), and cultured in BEGM with supplements according to the suppliers recommended protocol (Clonetics). Unless otherwise stated, all data was derived from experiments from 3 different HBEC sources. BEAS-2B cells (European Collection of Cell Cultures) were cultured in RPMI with 10% FCS (Invitrogen, Paisley, UK). HeLa cells were grown in DMEM with 10% FCS (Invitrogen), and used for RV titration assays. Major group RV16 and minor group RV1B were grown in HeLa cells, after three cycles of freeze and thawing, supernatant and cellular material where clarified by centrifugation at 4,000 rpm for 15 min, filter sterilized, aliquoted and frozen at -80°C. The serotype of all RV stocks was confirmed by titration with serotype specific anti-sera (American Type Culture Collection), and all RV stocks and cells were confirmed to be free of *Mycoplasma* contamination using a commercially available detection kit (Roche, Burgess Hill, UK).

### Transient transfections with siRNA or plasmid DNA

HBECs were cultured to 80% confluency in 12 well plates and transfected with 100 nM specific siRNA or control siRNA (specific for luciferase, Dharmacon, Lafayette, CO, USA), for 24 h prior to infection with RV1B. Time courses and dose responses of siRNA were performed previously to determine optimum conditions for knockdown of target genes. All siRNA achieved at least a 75% knockdown of target mRNA, and each siRNA was assessed for the induction of IFN or pro-inflammatory cytokine mRNA in the absence of infection. HBECs were cultured to near confluency in 12 well plates and then transfected with 0.25 µg per well of ΔTRIF-pUNO1 (a constitutive active TRIF cDNA, Invivogen, San Diego CA, USA), or pUNO1 control plasmid (Invivogen) or left untransfected for 5 h. All transfections were with Lipofectamine 2000 (Qiagen, Crawley, UK) according to the manufacturers recommended protocol. Complexes were removed, medium replaced and cells left for 24 h. RNA was then extracted and RIG-I, MDA5 mRNA measured. ΔRIG-I, RIG-IC and pEF-BOS control vector [24] were used to transfect BEAS-2B cells, at 0.25–0.5 µg per well, using Superfect (Qiagen) according to the manufacturer's recommended protocol.

### Infection of HBECs and BEAS-2B cells

HBECs were cultured to 80% confluency in 12 well plates, using BEGM (Clonetics) and starved in BEBM (no supplements) overnight and infected with RV1B for 1 h with shaking at room temperature, and samples taken at appropriate time points. Alternatively, HBECs were transfected with siRNA (Dharmacon), placed in BEGM without serum and then infected with RV1B. BEAS-2B cells were placed in 2% FCS containing RPMI medium overnight and infected with RV1B or RV16 for 1 h as above and placed in 2% FCS containing RPMI medium until required. For experiments involving enumerating virus replication, adhered virus was washed off by three additions of medium after the 1 h infection period.

### RNA extraction, cDNA synthesis and quantitative PCR

Total RNA was extracted from HBECs (RNeasy kit, Qiagen), and 2 µg was used for cDNA synthesis (Omniscript RT kit, Qiagen). Total RNA was also extracted from the upper left lobe of the mouse lung, and placed in RNA later (Qiagen), prior to RNA extraction and cDNA synthesis (as above). Quantitative PCR was carried out using specific primers and probes for each gene (Table S4 in Supporting Information S1). Reactions consisted of 12.5 µl of 2X Quanti-Tect Probe PCR Master Mix (Qiagen) in 25 µL. cDNA for 18S amplifications were diluted 1/100 in sterile water. Reactions were analyzed using an ABI 7000 TaqMan, (ABI Foster City, CA, USA) at 50°C for 2 min, 94°C for 10 min, and 45 cycles of 94°C for 15 s and 60°C for 15 s. Each gene was normalized to 18S rRNA, and for HBEC studies, presented as copies of each mRNA per 2×10^5^ cells, and for mouse lung, per cDNA reaction using a standard curve based on amplification with plasmid DNA. For siRNA experiments, copy number was expressed as a % of copy number versus control siRNA.

### SDS PAGE, Western blotting and immunofluorescence

For western blotting, total protein lysates were run on 4–12% Bis-Tris polyacrylamide gels, and transferred onto nitrocellulose membranes (Invitrogen), blocked in 5% skim milk, and probed with antibodies specific for mouse and human RIG-I (Cell Signaling, Danvers, MA, USA), diluted to 0.083 µg/mL, MDA5 1 µg/mL (Santa Cruz Biotechnology Inc, CA, USA), α-tubulin 0.2 µg/mL (Santa Cruz Biotechnology Inc), or β-actin, 1 µg/mL (Biovision, Mountain View CA, USA). Secondary antibodies used were goat anti-mouse HRP, 0.08 µg/mL, sheep anti-rabbit HRP, 2 µg/mL (AbD Serotec, Oxford, UK) and swine anti-goat HRP 1.4 µg/mL (Invitrogen). Blots were developed using ECL (GE Healthcare, Chalfont St Giles, UK). For immunohistochemistry, HBECs were grown on 8 well chamber slides (Nunc, Rochester NY, USA), infected with RV or treated with 10 ng/mL IFN-β, 5 µg/mL polyIC or medium for 8 or 24 h, and washed with PBS, fixed in 4% paraformaldehyde at room temperature for 5–7 min, washed once with PBS, and permeabilized with 0.2% Triton X-100 for 5 min at room temperature, and washed again in PBS. Slides were then blocked with a 1% BSA, 10% FCS-PBS overnight at 4°C. Cells were then stained with either anti-RIG-I, 2 µg/mL (Santa Cruz Biotechnology, Inc) or anti-MDA5, 2.7 µg/mL (Santa Cruz Biotechnology Inc) for 1 h at room temperature. Slides were washed three times with PBS, and stained with for 1 h at room temperature with donkey anti-goat Alexa Fluor 488, 6.7 µg/mL, and washed as above. Slides were then mounted with 4,6-diamidino-2-phenyindole dilactate containing mounting medium, and analysed using a colour CCD camera microscope (Zeiss, Rugby, UK).

### Analysis of human bronchial epithelium from individuals challenged with RV16 *in vivo*

Paraffin embedded bronchial biopsies were obtained from 15 non-asthmatic non-smoking individuals used in a previous *in vivo* challenge study with RV16 [16]. Samples were coded, and analyzed blind according to infection status, for RIG-I and MDA5 prior to infection (baseline) or at day 4 after infection. Goat antibodies to RIG-1 and MDA5 (Santa Cruz Biotechnology, Inc) at 2 µg/mL and 1 µg/mL were used respectively. Swine anti-goat LSAM-HRP reagents (DakoCytomation, Ely, UK), were used as per the manufacturer's recommended protocol, and antibody binding visualized using perioxidase staining. Staining intensity on surface epithelium was scored accordingly as 0–3, with no staining scored as 0 and intense staining scored as 3.

### Infection of *IFNAR1* deficient and wildtype mice with RV1B

Female *IFNAR1* ^−/−^ and 129/SvJ control mice aged 6–9 weeks were inoculated intranasally with 5×10^6^ TCID_50_ of RV1B, essentially as previously described [39], and culled humanely by lethal injection at various time points.

### Ethics statement

The human experimental challenge study was approved by St Mary's NHS Trust. All volunteers gave informed, written consent. All animal work was in accordance with Project License PPL70/6387, and performed according to regulations outlined by the Home Office, UK, in agreement with the Animals (Scientific Procedures) Act 1986.

### Statistical analysis

All *in vitro* experiments were performed 5–6 times, Figures 1, 3–7 used 3 independent HBEC donors, and data generated in the supporting information file also utilized 3 independent HBEC donors. For siRNA experiments, data from each independent experiment was converted to a % of the control siRNA + RV data, and mean ± SEM generated. All other data were expressed as mean ± SEM. Experiments using siRNA or transfection with plasmids were analyzed by one ANOVA and Bonferroni's multiple comparison test, and time course data using two-way ANOVA and Bonferroni's multiple comparison test, using GraphPad Prism software with *p*<0.05 taken as significant. For differences between two groups, a student's t-test was employed with *p*<0.05 taken as significant. Experiments in the mouse model involved 3–4 animals per group, in two independent experiments, (total of 6–8 animals) data were analyzed using two-way ANOVA and Bonferroni's multiple comparison test in GraphPad Prism. Staining of human bronchial epithelium for RIG-I and MDA5 was analyzed by using the paired Mann-Whitney U test, *p*<0.05 taken as significant.

## Supporting Information

Supporting Information S1 Tables S1 to S4.
(0.09 MB DOC)

Figure S1 Role of TRIF and Cardif in RV1B induced IFN gene expression in HBECs. (A) siRNA specific to TRIF and Cardif reduced RV1B induced IFN-β compared to control siRNA at 24h post infection. (B) siRNA specific to TRIF and Cardif reduced RV1B induced IFN-λ1 compared to control siRNA at 24h post infection. (C) siRNA specific to TRIF reduced RV1B induced IFN-λ2/3 compared to control siRNA however siRNA specific to Cardiff did not significantly reduce RV1B induced IFN-λ2/3 at 24h post infection. *p<0.05, ***p<0.001 versus control siRNA + RV1B, NS = not significant versus control siRNA+RV1B, n = 5 independent experiments, from 3 different HBEC donors, 2 experiments per donors 1,2 and one experiment for donor 3.
(10.10 MB TIF)

Figure S2 Role of TRIF and Cardif in RV1B induced pro-inflammatory cytokine gene expression in HBECs. (A) siRNA specific to TRIF and Cardif reduced RV1B induced rantes compared to control siRNA at 24h post infection. (B) siRNA specific to TRIF and Cardif reduced RV1B induced IP-10 compared to control siRNA at 24h post infection. (C) siRNA specific to TRIF and Cardif reduced RV1B induced IL-8 compared to control siRNA at 24h post infection. (D) siRNA specific to TRIF and Cardif reduced RV1B induced ENA-78 compared to control siRNA at 24h post infection. ***p<0.001 versus control siRNA+RV1B, NS  =  not significant versus control siRNA + RV1B, n = 5 independent experiments, from 3 different HBEC donors, 2 experiments per donors 1,2 and one experiment per donor 3.
(0.23 MB TIF)

Figure S3 RIG-I mediates the anti-viral responses in RV infection in BEAS-2B cells. (A). Constitutively active RIG-I, (ΔRIG-I) suppressed RV16 vRNA compared to pEF-BOS control at 24h post infection. (B). ΔRIG-I suppressed RV16 release compared to pEF-BOS control at 48h post infection. (C). ΔRIG-I suppressed RV1B vRNA compared to pEF-BOS control at 24h post infection. (D). ΔRIG-I suppressed RV16 release compared to pEF-BOS control at 48h post infection. *p<0.05, **p<0.01, ***p<0.001 as indicated n = 4-6 independent experiments.
(0.21 MB TIF)

Figure S4 Proposed model of sequential involvement of TLR3/TRIF and RIG-I/MDA5 in RV infecton. TLR3 and TRIF initially are involved in RV signalling in the recognition of RV infection and signal transduction within the endosome, and induce IFN-β, IFN-λ and RIG-I and MDA5 early with the infection cycle (4-12h). After 12h, increases in RIG-I and MDA5 protein in the intracellular compartment recognise a concominant increase in intracellular RV dsRNA and ssRNA. This process induces robust IFN-β and IFN-λ and possibly further RIG-I and MDA5 gene expression.
(6.49 MB TIF)
