# Correction: Co-ordinated Role of TLR3, RIG-I and MDA5 in the Innate Response to Rhinovirus in Bronchial Epithelium

**DOI:** 10.1371/annotation/c3f58c59-a42f-495b-a4f2-d74d55d72166

**Published:** 2012-04-03

**Authors:** Louise Slater, Nathan W. Bartlett, Jennifer J. Haas, Jie Zhu, Simon D. Message, Ross P. Walton, Annemarie Sykes, Samer Dahdaleh, Deborah L. Clarke, Maria G. Belvisi, Onn M. Kon, Takashi Fujita, Peter K. Jeffery, Sebastian L. Johnston, Michael R. Edwards

The following information was missing from the funding section: This work was funded in part by the MoRIAE European Research Council FP7 Advanced Investigator award to SLJ, Grant number 233015.

