# Supplementary material for: Co-ordinated Role of TLR3, RIG-I and MDA5 in the Innate Response to Rhinovirus in Bronchial Epithelium
Source: PLoS Pathog. 2010 Nov 4;6(11):e1001178. doi: 10.1371/journal.ppat.1001178 (PMC2973831; doi:10.1371/journal.ppat.1001178)
Supplement: Supporting Information S1 — Tables S1 to S4. (0.09 MB DOC) [file ppat.1001178.s001.doc]

**Supporting Information S1**

**Table S1. Effects of the TLR7/8 agonist R848 on bronchial HBEC IFN-β, IFN-λ and RIG-I, MDA5 mRNA expression.**

| **Effects of R848 on IFN and RIG-I and MDA5 gene expression** | | | | | | |
| --- | --- | --- | --- | --- | --- | --- |
| **8h** | | | | **24h** | | |
|  | **medium** | **R848**  **1 μM** | **R848**  **10 μM** | **medium** | **R848**  **1 μM** | **R848**  **10 μM** |
| **IFN-β** | 2.7x10^3^  ± 8.6x10^2^ | 9.9x10^3^  ± 5.3x10^3^  NS | 1.5x10^4^  ±1.2x10^3^  NS | 4.0x10^3^  ± 2.6x10^3^ | 1.9x10^4^  ± 1.2x10^4^  NS | 1.8x10^4^  ±3.2x10^3^  NS |
| **IFN-λ1** | 17.0  ± 5.8 | 81.8  ± 64.9  NS | ND | 11.5  ± 1.8 | 4.6  ± 2.7  NS | ND |
| **IFN-λ2/3** | 1.2x10^3^  ± 9.0x10^2^ | 8.1  ± 5.0  NS | ND | 3.2x10^2^  ± 2.6x10^2^ | 1.7  ± 1.5  NS | ND |
| **RIG-I** | 2.6x10^5^  ± 8.7x10^4^ | 8.4x10^5^  ± 5.7x10^5^  NS | ND | 7.8x10^5^  6.5x10^5^ | 1.3x10^6^  8.6x10^5^  NS | ND |
| **MDA5** | 6.5x10^4^  ± 7.0x10^3^ | 8.4x10^4^  ± 8.1x10^3^  NS | ND | 1.2x10^5^  ± 4.3x10^4^ | 9.6x10^4^  ± 2.3x10^4^  NS | ND |
|  |  |  |  |  |  |  |

NS=non-significant versus medium treated controls. *n*=3-4 independent experiments, from 2 different HBEC donors. ND=no data.

**Table S2. Effects of specific siRNA on IFN gene expression in the absence of RV infection.**

| **Mean ± SEM mRNA Copy Number per Realtime PCR Reaction** | | | |
| --- | --- | --- | --- |
|  | **IFN-β** | **IFN-λ1** | **IFN-λ2/3** |
| **Untransfected** | 5.8x10^2^ ± 5.1x10^2^ | 1.0x10^2^ ± 5.8x10^1^ | 1.3x10^3^ ± 6.7x10^2^ |
| **Oligo dsDNA^†^** | 2.5x10^2^± 2.1x10^2^  NS | 2.2x10^3^ ± 1.5x10^3^  NS | 1.9x10^3^ ± 1.0x10^3^  NS |
| **Control siRNA** | 7.7x10^1^ ± 2.3x10^1^  NS | 9.1x10^2^ ± 5.3x10^2^  NS | 2.3x10^3^ ± 1.3x10^3^  NS |
| **RIG-I siRNA** | 9.3x10^2^ ± 4.8x10^2^  NS | 1.3x10^1^ ± 0.1x10^1^  NS | 6.5x10^2^ ± 4.5x10^2^  NS |
| **MDA5 siRNA** | 3.0x10^2^ ± 2.6x10^2^  NS | 6.3x10^2^ ± 5.8x10^2^  NS | 1.8x10^3^ ± 1.3x10^3^  NS |
| **TLR3 siRNA** | 4.2x10^1^ ± 0.5x10^1^  NS | 1.1x10^2^ ± 9.7x10^1^  NS | 7.0x10^2^ ± 6.5x10^2^  NS |
| **TRIF siRNA** | 4.2x10^1^ ± 0.5x10^1^  NS | 0.1x10^1^ ± 0.1x10^1^  NS | 3.2x10^2^ ± 2.2x10^2^  NS |
| **Cardif siRNA** | 1.1x10^3^ ± 7.0x10^2^  NS | 4.0x10^2^ ± 3.5x10^2^  NS | 1.1x10^3^ ± 9.3x10^2^  NS |

^†^To monitor the effects of transfection, cells were transfected with a non-specific dsDNA oligonucleotide corresponding to the AP-1 binding site of the human IL-8 promoter. NS=non-significant versus untransfected controls. *n*=4-6 independent experiments, from 3 different HBEC donors were performed. Two experiments per donor were performed for IFN-β (*n*=6 experiments), while for IFN-λ1 and IFN-λ2/3 measurements; 2 experiments for donor 1 and 1 experiment each for donor 2 and 3 were performed ( a total of *n*=4 experiments).

**Table S3. Effects of specific siRNA on pro-inflammatory cytokine gene expression in the absence of RV infection.**

| **Mean ± SEM mRNA Copy Number per Realtime PCR Reaction** | | | | |
| --- | --- | --- | --- | --- |
|  | **Rantes** | **IP-10** | **IL-8** | **ENA-78** |
| **Untransfected** | 5.4x10^5^ ± 3.8x10^5^ | 1.8x10^6^ ± 9.0x10^5^ | 3.5x10^6^ ± 1.0x10^6^ | 3.2x10^6^ ± 6.7x10^4^ |
| **Oligo dsDNA^†^** | 1.0x10^6^ ± 4.0x10^5^  NS | 2.5x10^6^ ± 9.1x10^5^  NS | 3.9x10^6^ ± 1.0x10^6^  NS | 7.8x10^5^ ± 2.1x10^6^  NS |
| **Control siRNA** | 4.9x10^5^ ± 1.2x10^5^  NS | 2.3x10^6^ ± 3.1x10^5^  NS | 4.8x10^6^ ± 4.8x10^5^  NS | 1.3x10^6^ ± 2.4x10^5^ |
| **RIG-I siRNA** | 1.4x10^5^ ± 9.1x10^4^  NS | 4.8x10^5^ ± 2.0x10^5^  NS | 3.3x10^6^ ± 6.9x10^5^  NS | 2.9x10^5^ ± 1.3x10^5^  NS |
| **MDA5 siRNA** | 1.8x10^5^ ± 9.7x10^4^  NS | 6.1x10^5^ ± 2.3x10^5^  NS | 3.2x10^6^ ± 1.0x10^6^  NS | 3.7x10^5^ ± 1.0x10^5^  NS |
| **TLR3 siRNA** | 9.3x10^4^ ± 5.9x10^4^  NS | 5.5x10^5^ ± 2.4x10^5^  NS | 1.7x10^6^ ± 4.8x10^5^  NS | 2.6x10^5^ ± 1.2x10^5^  NS |
| **TRIF siRNA** | 2.1x10^5^ ± 1.3x10^6^  NS | 9.3x10^5^ ± 2.8x10^6^  NS | 4.4x10^6^ ± 1.9x10^6^  NS | 2.5x10^5^ ± 1.0x10^5^  NS |
| **Cardif siRNA** | 3.4x10^5^ ± 2.9x10^5^  NS | 1.9x10^5^ ± 7.1x10^4^  NS | 3.9x10^6^ ± 1.4x10^6^  NS | 1.2x10^5^ ± 7.0x10^4^  NS |

^†^To monitor the effects of transfection, cells were transfected with a non-specific dsDNA oligonucleotide corresponding to the AP-1 binding site of the human IL-8 promoter. NS=non-significant versus untransfected controls. *n*=4 independent experiments from 3 different HBEC donors; 2 experiments for donor 1 and 1 experiment each for donor 2 and 3.

**Table S4. Primer and Probe sequences for use in Realtime RT-PCR.**

| **Gene** | **Primer/**  **Probe** | **Sequence (5’-3’)** |
| --- | --- | --- |
| **IFN-β**  **NM_002176** | Forward  Reverse  Probe | CGCCGCATTGACCATCTA  TTAGCCAGGAGGTTCTCAACAATAGTCTCA  FAM-TCAGACAAGATTCATCTAGCACTGGCTGGA-TAMRA |
| **IFN-λ1**  **NM_172140** | Forward  Reverse  Probe | GGACGCCTTGGAAGAGTCACT  AGAAGCCTCAGGTCCCAATTC  FAM-AGTTGCAGCTCTCCTGTCTTCCCCG-TAMRA |
| **IFN-λ2/3**  **NM_172139** | Forward  Reverse  Probe | CTGCCACATAGCCCAGTTCA  AGAAGCGACTCTTCTAAGGCATCTT  FAM-TCTCCACAGGAGCTGCAGGCCTTTA-TAMRA |
| **IL-8**  **NM_000584** | Forward  Reverse  Probe | ctggccgtggctctcttg  ccttggcaaaactgcacctt  FAM-cagccttcctgatttctgcagctctgtgt-TAMRA |
| **ENA-78**  **NM_002994** | Forward  Reverse  Probe | agagctgcgttgcgtttgt  tggcgaacacttgcagattact  FAM-acagaccacgcaaggagttcatccca-TAMRA |
| **Rantes**  **NM_002985** | Forward  Reverse  Probe | gcatctgcctccccatattc  cagtgggcgggcaatg  FAM-tcggacaccacaccctgctgct-TAMRA |
| **IP-10**  **NM_001565** | Forward  Reverse  Probe | cCattctgatttgctgccttatc  gcaggtacagcgtacagttct  FAM-ctgactctaagtggcattcaaggagtacctc  tctc-TAMRA |
| **RIG-I**  **NM_014314** | Forward  Reverse  Probe | CCAAGCCAAAGCAGTTTTCAAG  CACATGGATTCCCCAGTCATG  FAM-TTGAAAAAAGAGCAAAGATATTCTGTGCCCG  AC-TAMRA |
| **MDA5**  **NM_022168** | Forward  Reverse  Probe | GATTCAGGCACCATGGGAAGT  AGGCCTGAGCTGGAGTTCTG  FAM-GGGATGCTCTTGCTGCCACATTCTCTT-TAMRA |
| **TLR3**  **NM-003265** | Forward  Reverse  Probe | AAATTAAAGAGTTTTCTCCAGGGTGTT  ATTCCGAATGCTTGTGTTTGC  FAM-TTTGGCCTCTTTCTGAACAATGTCCAGC-TAMRA |
| **TRIF**  **NM_014261** | Forward  Reverse  Probe | TGCACAGGCCCATCACTTC  AGTTTGTGCTTCAGATACAAGAGCTT  FAM-TAGCGCCTTCGACATTCTAGGTGCAGC-TAMRA |
| **RV**  **In house** | Forward  Reverse  Probe | gtgaagagccscrtgtgct  gctscagggttaaggttagcc  FAM-tgagtcctccggcccctgaatg-TAMRA |
| **Mouse**  **IFN-β**  **NM_010510** | Forward  Reverse  Probe | ccatcatgaacaacaggtggat  gagagggctgtggtggagaa  FAM-ctccacgctgcgttcctgctgtg-TAMRA |
| **Mouse**  **IFN-λ**  **In house** | Forward  Reverse  Probe | aaaggattgccacattgctc  tcaagcagcctctttctcgat  FAM-ccccaaaagagctgcaggc-TAMRA |
| **Mouse RIG-I**  **NM_172689** | Forward  Reverse  Probe | gggcgtggcagaaca  gcctccagaacacacttctgt  FAM-ccgggcaacaggaatgacgct-TAMRA |
| **Mouse MDA5**  **EF113547** | Forward  Reverse  Probe | ttgacagtcgaagacagaaatcg  gcagctctcttacacctgactcatt  FAM-ttctgctgcaggaaacagcgg-TAMRA |
| **18S rRNA**  **M10098** | Forward  Reverse  Probe | cgccgctagaggtgaaattct  cattcttggcaaatgctttcg  FAM-accggcgcaagacggaccaga-TAMRA |
