# Supplementary figures and images for: Co-ordinated Role of TLR3, RIG-I and MDA5 in the Innate Response to Rhinovirus in Bronchial Epithelium
Source: PLoS Pathog. 2010 Nov 4;6(11):e1001178. doi: 10.1371/journal.ppat.1001178 (PMC2973831; doi:10.1371/journal.ppat.1001178)

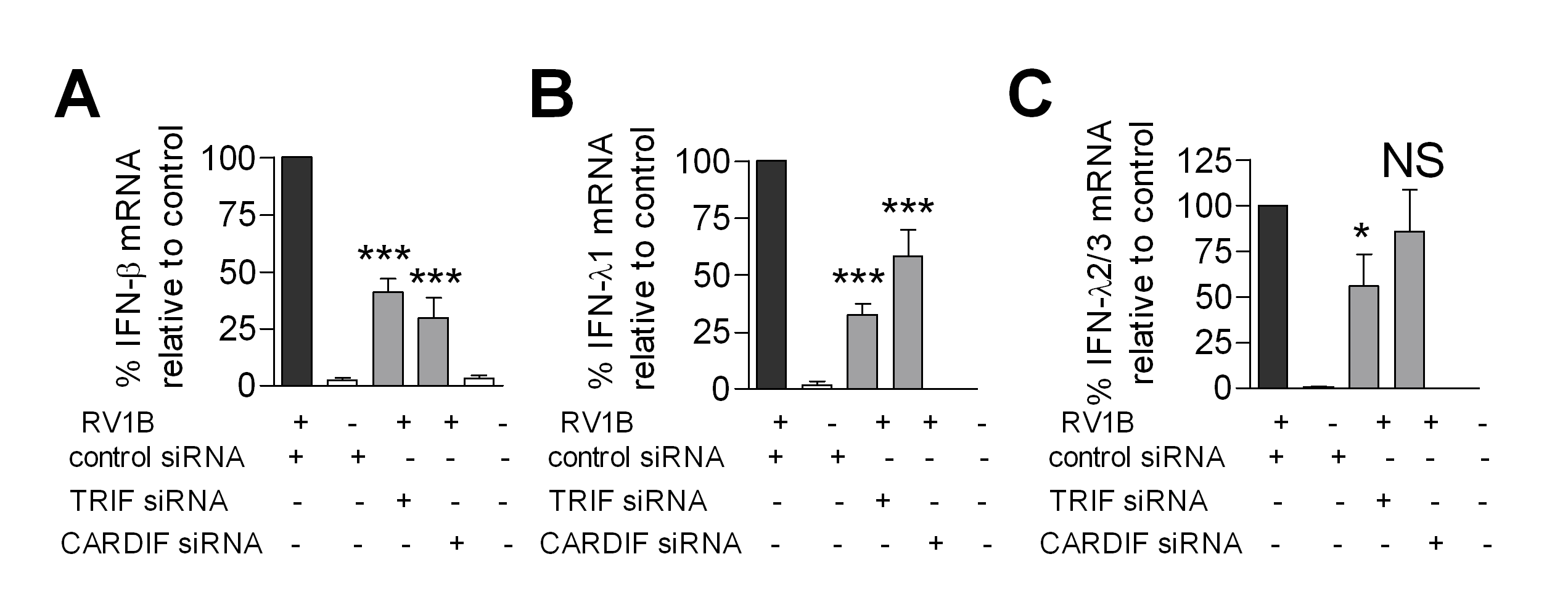

Supplement: Figure S1 — Role of TRIF and Cardif in RV1B induced IFN gene expression in HBECs. (A) siRNA specific to TRIF and Cardif reduced RV1B induced IFN-β compared to control siRNA at 24h post infection. (B) siRNA specific to TRIF and Cardif reduced RV1B induced IFN-λ1 compared to control siRNA at 24h post infection. (C) siRNA specific to TRIF reduced RV1B induced IFN-λ2/3 compared to control siRNA however siRNA specific to Cardiff did not significantly reduce RV1B induced IFN-λ2/3 at 24h post infection. *p<0.05, ***p<0.001 versus control siRNA + RV1B, NS = not significant versus control siRNA+RV1B, n = 5 independent experiments, from 3 different HBEC donors, 2 experiments per donors 1,2 and one experiment for donor 3. (10.10 MB TIF) [file ppat.1001178.s002.tif]

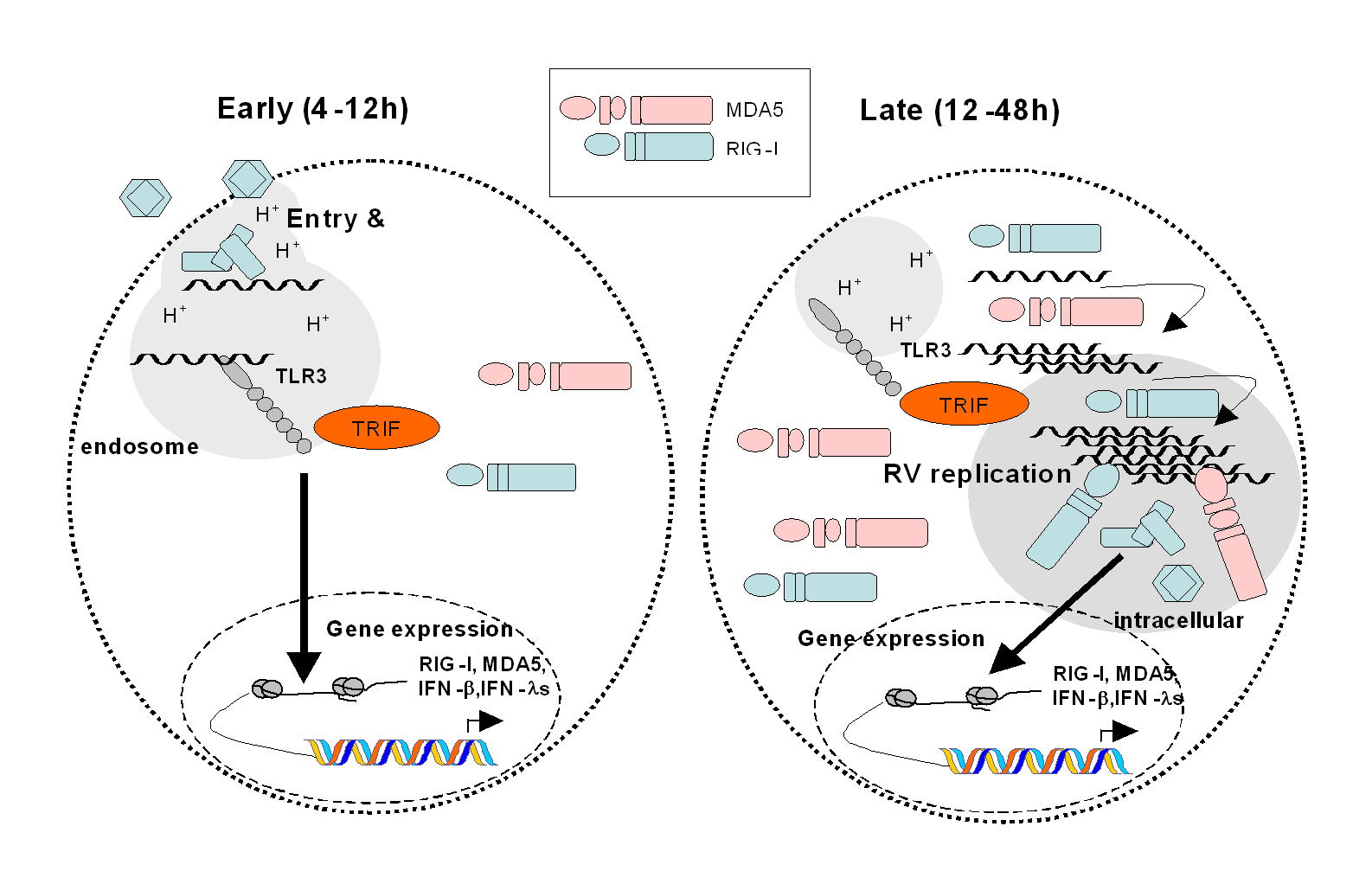

Supplement: Figure S4 — Proposed model of sequential involvement of TLR3/TRIF and RIG-I/MDA5 in RV infecton. TLR3 and TRIF initially are involved in RV signalling in the recognition of RV infection and signal transduction within the endosome, and induce IFN-β, IFN-λ and RIG-I and MDA5 early with the infection cycle (4-12h). After 12h, increases in RIG-I and MDA5 protein in the intracellular compartment recognise a concominant increase in intracellular RV dsRNA and ssRNA. This process induces robust IFN-β and IFN-λ and possibly further RIG-I and MDA5 gene expression. (6.49 MB TIF) [file ppat.1001178.s005.tif]
